# Supplementary material for: The impact of antenatal syphilis point of care testing on pregnancy outcomes: A systematic review
Source: PLoS One. 2021 Mar 25;16(3):e0247649. doi: 10.1371/journal.pone.0247649 (PMC7993761; doi:10.1371/journal.pone.0247649)
Supplement: S1 Fig — (A) Laboratory testing algorithms for the diagnosis of syphilis. RPR, rapid plasma reagin. VDLR, veneral disease research laboratory. TPHA, treponema pallidum heamoagglutination assay. TPPA, treponema pallidum particle agglutination [62, 63]. (B) Point-of-care testing algorithms for the diagnosis of syphilis. ICS, immunochromatographic strip. POCT, point-of-care testing. RPR, rapid plasma reagin [63]. (DOCX) [file pone.0247649.s002.docx]

**Reverse**

**algorithm**

**Traditional**

**algorithm**

Treponemal

Test

*TPHA, TPPA*

Nontreponemal

Test

*RPR, VDRL test*

Screening

-

+

+

+

-

+v

+

+

Nontreponemal

Test

*RPR or other*

Treponemal

Test

*TPHA or other*

Confirmation

-

No Syphilis

+

Syphilis

-

No Syphilis

+

Syphilis

Treatment at

first follow-up

visit

Treatment at

first follow-up

visit

Treatment

S1 Fig. (A) Laboratory testing algorithms for the diagnosis of syphilis

RPR, rapid plasma reagin. VDLR, veneral disease research laboratory. TPHA, treponema pallidum heamoagglutination assay. TPPA, treponema pallidum particle agglutination. [62, 63]

Nontreponemal

POCT

*Onsite RPR*

Treponemal POCT

*ICS*

S1 Fig. (B) Point-of-care testing algorithms for the diagnosis of syphilis

ICS, immunochromatographic strip. POCT, point-of-care testing. RPR, rapid plasma reagin. [62]

Same day

treatment

Same day

treatment

Same day

treatment

**Dual**

**POCT**

Dual treponemal/

Nontreponemal

POCT

*DPP*

-

No syphilis

+

Syphilis

-

No syphilis

-

No syphilis

+

Syphilis

(past or present)

**Nontreponemal**

**POCT**

+

Syphilis

**Treponemal**

**POCT**

Screening

Treatment
