# Supplementary material for: The impact of antenatal syphilis point of care testing on pregnancy outcomes: A systematic review
Source: PLoS One. 2021 Mar 25;16(3):e0247649. doi: 10.1371/journal.pone.0247649 (PMC7993761; doi:10.1371/journal.pone.0247649)
Supplement: S1 Table — Based on searches last conducted on June 8, 2020 in PubMed, Medline (Ovid) and Cochrane. (DOCX) [file pone.0247649.s003.docx]

S1 Table. Search strategies and hits based on searches last conducted on June 8, 2020 in PubMed, Medline (Ovid) and Cochrane

| **Database** | **Search strategy** | **Number hits** |
| --- | --- | --- |
| PubMed | (((((((syphilis[MeSH Terms]) OR (treponema pallidum[MeSH Terms])) OR (syphilis)) OR (treponema pallidum)) OR (syphilis infected women)) AND ((((((point-of-care testing[MeSH Terms]) OR (point-of-care systems[MeSH Terms])) OR (point-of-care testing)) OR (point-of-care diagnostics)) OR (point-of-care systems)) OR (rapid testing))) AND ((((((Pregnancy[MeSH Terms]) OR (Pregnancy)) OR (Pregnant)) OR (Antenatal)) OR (Prenatal)) OR (Pregnant women))) AND (((((((((((((((((((((((((((pregnancy outcome[MeSH Terms]) OR ("Syphilis, Congenital"[Mesh])) OR (stillbirth[MeSH Terms])) OR (Perinatal death[MeSH Terms])) OR (infant, low birth weight[MeSH Terms])) OR (fetal death[MeSH Terms])) OR (Infant premature[MeSH Terms])) OR (Mortality[MeSH Terms])) OR (Death[MeSH Terms])) OR (abortion, spontaneous[MeSH Terms])) OR (Pregnancy complications[MeSH Terms])) OR (Pregnancy outcome)) OR (Congenital syphilis)) OR (Stillbirth)) OR (Perinatal death)) OR (Low birth weight)) OR (fetal death))) OR (prematurity)) OR (Mortality)) OR (Death)) OR (Spontaneous abortion)) OR (Pregnancy complications)) OR (Clinical evidence of syphilis)) OR (Neonatal death)) OR (Infant death)) OR (Perinatal mortality)) | 241 |
| Medline (Ovid) | (exp Syphilis/ or syphilis*.mp. or exp Treponema pallidum/ or treponema pallidum*.mp. or syphilis infected women*.mp.) AND (exp Point-of-Care Testing/ or exp Point-of-Care Systems/ or point-of-care testing*.mp. or point-of-care systems*.mp. or point of care diagnostics.mp. or rapid testing*.mp.) AND (exp Pregnancy/ or Pregnancy*.mp. or Pregnant*.mp. or Antenatal*.mp. or Prenatal*.mp. or Pregnant women*.mp.) AND (exp Pregnancy Outcome/ or exp Syphilis, Congenital/ or exp Stillbirth/ or exp Perinatal Death/ or exp Infant, Low Birth Weight/ or exp Fetal Death/ or exp Infant, Premature/ or exp Mortality/ or exp Death/ or exp Abortion, Spontaneous/ or exp Pregnancy Complications/ or pregnancy outcome*.mp. or congenital syphilis*.mp. or stillbirth*.mp. or perinatal death*.mp. or low birth weight*.mp. or fetal death*.mp. or prematurity*.mp. or mortality*.mp. or death*.mp. or spontaneous abortion*.mp. or pregnancy complications*.mp. or clinical evidence of syphilis*.mp. or neonatal death*.mp. or infant death*.mp. or perinatal mortality*.mp) | 58 |
| Cochrane | (MeSH descriptor: [Syphilis] explode all trees OR MeSH descriptor: [Treponema pallidum] explode all trees OR (syphilis):ti,ab,kw OR ("Treponema pallidum"):ti,ab,kw OR (syphilis infected women):ti,ab,kw) AND (MeSH descriptor: [Point-of-Care Systems] explode all trees OR MeSH descriptor: [Point-of-Care Testing] explode all trees OR ("point of care testing"):ti,ab,kw OR (point-of-care systems):ti,ab,kw OR (point-of-care diagnostics):ti,ab,kw OR (rapid testing):ti,ab,kw) AND (MeSH descriptor: [Pregnancy] explode all trees OR ("pregnancy"):ti,ab,kw OR (pregnant):ti,ab,kw OR (antenatal):ti,ab,kw OR (prenatal):ti,ab,kw OR (pregnant women):ti,ab,kw )AND (MeSH descriptor: [Pregnancy Outcome] explode all trees OR MeSH descriptor: [Syphilis, Congenital] explode all trees OR MeSH descriptor: [Stillbirth] explode all trees OR MeSH descriptor: [Perinatal Death] explode all trees OR MeSH descriptor: [Infant, Low Birth Weight] explode all trees OR MeSH descriptor: [Fetal Death] explode all trees OR MeSH descriptor: [Infant, Premature] explode all trees OR MeSH descriptor: [Mortality] explode all trees OR MeSH descriptor: [Death] explode all trees OR MeSH descriptor: [Abortion, Spontaneous] explode all trees OR MeSH descriptor: [Pregnancy Complications] explode all trees OR (pregnancy outcome):ti,ab,kw OR (congenital syphilis):ti,ab,kw OR (stillbirth):ti,ab,kw OR (perinatal death):ti,ab,kw OR ("low birth-weight"):ti,ab,kw OR (fetal death):ti,ab,kw OR (prematurity):ti,ab,kw OR (mortality):ti,ab,kw OR (death):ti,ab,kw OR ("spontaneous abortion"):ti,ab,kw OR (pregnancy complications):ti,ab,kw OR (clinical evidence of syphilis):ti,ab,kw OR (neonatal death):ti,ab,kw OR (infant death):ti,ab,kw OR (perinatal mortality):ti,ab,kw) | 28 |
