# Supplementary material for: The impact of antenatal syphilis point of care testing on pregnancy outcomes: A systematic review
Source: PLoS One. 2021 Mar 25;16(3):e0247649. doi: 10.1371/journal.pone.0247649 (PMC7993761; doi:10.1371/journal.pone.0247649)
Supplement: S2 Table — (DOCX) [file pone.0247649.s004.docx]

S2 Table. Questions used for the critical appraisal for economic evaluations and randomized controlled trials as provided by Joanna Briggs Institute Reviewer´s Manual [30]

| Question numbers | Economic Evaluation | Randomized controlled trial |
| --- | --- | --- |
| 1 | Is there a well-defined question? | Was true randomization used for assignment of participants to treatment groups? |
| 2 | 1. Is there comprehensive description of alternatives? | Was allocation to treatment groups concealed? |
| 3 | Are all important and relevant costs and outcomes for each alternative identified? | Were treatment groups similar at the baseline? |
| 4 | Has clinical effectiveness been established? | Were participants blind to treatment assignment? |
| 5 | Are costs and outcomes measured accurately? | Were those delivering treatment blind to treatment assignment? |
| 6 | Are costs and outcomes valued credibly? | Were outcomes assessors blind to treatment assignment? |
| 7 | Are costs and outcomes adjusted for differential timing? | Were treatment groups treated identically other than the intervention of interest? |
| 8 | Is there an incremental analysis of costs and consequences? | Was follow up complete and if not, were differences between groups in terms of their follow up adequately described and analyzed? |
| 9 | Were sensitivity analyses conducted to investigate uncertainty in estimates of cost or consequences? | Were participants analyzed in the groups to which they were randomized? |
| 10 | Do study results include all issues of concern to users? | Were outcomes measured in the same way for treatment groups? |
| 11 | Are the results generalizable to the setting of interest in the review? | Were outcomes measured in a reliable way? |
| 12 | Not applicable | Was appropriate statistical analysis used? |
| 13 | Not applicable | Was the trial design appropriate, and any deviations from the standard RCT design (individual randomization, parallel groups) accounted for in the conduct and analysis of the trial? |
