# Supplementary material for: The impact of antenatal syphilis point of care testing on pregnancy outcomes: A systematic review
Source: PLoS One. 2021 Mar 25;16(3):e0247649. doi: 10.1371/journal.pone.0247649 (PMC7993761; doi:10.1371/journal.pone.0247649)
Supplement: S3 Table — Q: Questions based on the JBL risk assessment (Appendix 3). ✓: Indicates yes (1 point). Ο: Indicates No (0 points). ‘?’: Indicates unclear (0,5 points). Riskb: The risk of bias was considered high when the study score ≤ 49%, moderate when the study score reached 50 to 69%, and low when the study score reached ≥ 70%. N/A = not applicable. (DOCX) [file pone.0247649.s005.docx]

S3 Table. Results of the Joanna Briggs Institute critical appraisal checklist. Q: questions based on the JBL risk assessment (Appendix 3). ✓: Indicates yes (1 point). 🗴: Indicates No (0 points). ‘?’: Indicates unclear (0,5 points). Risk^b^: The risk of bias was considered high when the study score ≤ 49%, moderate when the study score reached 50 to 69%, and low when the study score reached ≥ 70%. N/A = not applicable.

| **Study** | **Q1** | **Q2** | **Q3** | **Q4** | **Q5** | **Q6** | **Q7** | **Q8** | **Q9** | **Q10** | **Q11** | **Q12** | **Q13** | | **% yes** | | | **Risk^b^** |
| --- | --- | --- | --- | --- | --- | --- | --- | --- | --- | --- | --- | --- | --- | --- | --- | --- | --- | --- |
| *Cost-effectiveness analysis* |  |  |  |  |  |  |  |  |  |  |  |  |  |  | |  | | |
| Bristow et al. (2016) (40) | 🗸 | 🗴 | 🗸 | 🗸 | 🗸 | 🗸 | 🗴 | 🗸 | 🗸 | 🗴 | 🗸 | N/A | N/A | 72%  8/11 | | | low | |
| Kuznik et al. (2015) (42) | 🗸 | 🗴 | 🗴 | 🗸 | 🗸 | 🗸 | 🗴 | 🗸 | 🗸 | 🗴 | 🗸 | N/A | N/A | 64%  7/11 | | | moderate | |
| Kuznik et al. (2013) (41) | 🗸 | 🗴 | 🗴 | 🗸 | 🗸 | 🗸 | 🗴 | 🗸 | 🗸 | 🗴 | 🗸 | N/A | N/A | 64%  7/11 | | | moderate | |
| Schackman et al. (2007) (43) | 🗸 | 🗴 | 🗸 | 🗸 | 🗸 | 🗸 | 🗴 | 🗸 | 🗸 | 🗸 | 🗸 | N/A | N/A | 81%  9/11 | | | low | |
| Owuso-Edusei et al. (2011) (46) | 🗴 | 🗸 | 🗸 | 🗸 | 🗸 | 🗸 | 🗴 | 🗸 | 🗸 | 🗸 | 🗸 | N/A | N/A | 81%  9/11 | | | low | |
| Rydzak et al. (2008) (45) | 🗸 | 🗸 | 🗴 | 🗸 | 🗸 | 🗸 | 🗴 | 🗸 | 🗸 | 🗸 | 🗸 | N/A | N/A | 81%  9/11 | | | low | |
| Blandford et al. (1007) (44) | 🗸 | 🗸 | 🗴 | 🗸 | 🗸 | 🗸 | 🗴 | 🗸 | 🗸 | 🗸 | 🗸 | N/A | N/A | 81%  9/11 | | | low | |
| Randomized controlled trial |  |  |  |  |  |  |  |  |  |  |  |  |  |  | | |  | |
| Munkhuu et al. (2009) (39) | ? | N/A | 🗸 | N/A | N/A | 🗴 | 🗸 | 🗸 | 🗸 | 🗸 | 🗸 | 🗸 | 🗸 | 85%  8,5/10 | | | low | |
| Myer et al. (2003) (38) | 🗸 | N/A | 🗸 | N/A | N/A | 🗴 | ? | 🗸 | 🗸 | 🗸 | 🗸 | 🗸 | 🗸 | 85%  8,5/10 | | | low | |
