# Supplementary material for: The impact of antenatal syphilis point of care testing on pregnancy outcomes: A systematic review
Source: PLoS One. 2021 Mar 25;16(3):e0247649. doi: 10.1371/journal.pone.0247649 (PMC7993761; doi:10.1371/journal.pone.0247649)
Supplement: Attachment — Submitted filename: Replies to reviewers comments.23.1.docx [file pone.0247649.s006.docx]

Review Comments to the Author

Please use the space provided to explain your answers to the questions above. You may also include additional comments for the author, including concerns about dual publication, research ethics, or publication ethics. (Please upload your review as an attachment if it exceeds 20,000 characters)

Reviewer #1: please see comments below- no comments for the authors at this time
I have significant concerns about the study design. The authors selected 2 clinical studies with different outcomes and 7 modeling studies for the analysis. I would suggest a statistical review for this. I don't believe they should use CEA or modeling studies for a systematic review. The two clinical studies had different outcomes that were measured, so I am not sure a meta-synthesis is appropriate. I will be willing to re-review the manuscript if a formal statistical review suggests this is acceptable scientific method.

Answer: We thank the reviewer for taking the time to review our work and provide us with insightful feedback.

The reviewer highlighted two points:

1) inclusion of studies with different clinical outcomes;

2) inclusion of modeling studies or CEA.

1) Considering the clinical implications of syphilis on expectant mothers and their babies can be diverse, we believed it relevant to capture, as much as possible, the full extent of such implications. We therefore decided to include all studies that reported on pregnancy outcomes in the context of point of care antenatal testing.

We would like to suggest that this is not an unusual approach for systematic reviews. This is particularly so in case of pregnancy outcomes, which can be quite diverse, but still relevant to be investigated. In fact, this if often the usual approach, as supported by several publications. To give just a few examples:

- The Association Between Vaginal Microbiota Dysbiosis, Bacterial Vaginosis, and Aerobic Vaginitis, and Adverse Pregnancy Outcomes of Women Living in Sub-Saharan Africa: A Systematic Review. Naomi C. A. Juliana, Meghan J. M. Suiters, Salwan Al-Nasiry, Servaas A. Morré, Remco P. H. Peters and Elena Ambrosino. Front. Public Health (2020);
- Advanced maternal age and adverse pregnancy outcomes: A systematic review and meta-analysis. Samantha C. Lean, Hayley Derricott,Rebecca L. Jones, Alexander E. P. Heazell. PLOS one (2017);
- Hall JA, Benton L, Copas A, Stephenson J. Pregnancy Intention and Pregnancy Outcome: Systematic Review and Meta-Analysis. Matern Child Health J (2017);
- Antiretroviral Therapy and Pregnancy Outcomes in Developing Countries: A Systematic Review. Alemu FM, Yalew AW, Fantahun M, Ashu EE Int J MCH AIDS (2015).

2) The purpose of systematic reviews is to synthesize the published scientific literature around a specific topic. However, there is often not enough evidence on a topic. In such instances, as suggested by Kuntz K et al. (Decision and Simulation Modeling in Systematic Reviews. Use of Modeling in Systematic Reviews: The EPC Perspective (2013) <https://www.ncbi.nlm.nih.gov/books/NBK127484/>), published modeling studies will provide added value in the context of systematic reviews.

The point raised by the reviewer was specifically addressed by Kuntz et al., with the goal to reach a consensus on the use of modeling evidence in the context of systematic reviews. The publication also stressed that ‘models are well-suited to address gaps in the literature and to synthesize literature from differing sources and contexts into a single representation of the empirical evidence’.

Further, previous literature suggests that adverse pregnancy outcomes associated with maternal syphilis infection can be reliably predicted from published data on disease prevalence, ANC coverage, treatment rates and screening and testing effectiveness (Newman L et al. Global estimates of syphilis in pregnancy and associated adverse outcomes: analysis of multinational antenatal surveillance data. PLoS Med (2013); Kuznik A et al. Estimating the Public Health Burden Associated With Adverse Pregnancy Outcomes Resulting From Syphilis Infection Across 43 Countries in Sub-Saharan Africa. Sex Transm Dis (2015)). All these input parameters were used in the included studies. This further supports the reliability of evidence from modeling studies.

Finally, previously published systematic reviews have included modelling studies or CEA. To give some examples:

- A Systematic Review of the Associations Between Inverse Dynamics and Musculoskeletal Modeling to Investigate Joint Loading in a Clinical Environment. Jana Holder, Ursula Trinler, Andrea Meurer and Felix Stief. Front. Bioeng. Biotechnol (2020)
- Systematic review of clinical prediction models to support the diagnosis of asthma in primary care. Daines L, McLean S, Buelo A, Lewis S, Sheikh A, Pinnock H. NPJ Prim Care Respir Med (2019)
- A systematic review of the cost-effectiveness of medication adherence-enhancing intervention for asthma. Sin Mei Khaw, Shu Chuen Li, Nurul Ain Mohd Tahir. J Asthma (2021)
- Cost-effectiveness of Pneumococcal Vaccines Among Adults Over 50 Years Old in Low- and Middle-Income Countries: A Systematic Review. Yixue Shao, Charles Stoecker. Expert Rev Vaccines (2021)
- Examining Equity Effects of Health Interventions in Cost-Effectiveness Analysis: A Systematic Review. Anton L V Avanceña, Lisa A Prosser. Value Health (2021)
- An Updated Systematic Review of Cost-Effectiveness Analyses of Drugs for Osteoporosis. Nannan Li, Dennis Cornelissen, Stuart Silverman, Daniel Pinto, Lei Si, Ingrid Kremer, Sandrine Bours, Robin de Bot, Annelies Boonen, Silvia Evers, Joop van den Bergh, Jean-Yves Reginster, Mickaël Hiligsmann. Pharmacoeconomics (2020)

Reviewer #2: This is an excellent systemic review of studies on the impact of antenatal syphilis point of care testing on pregnancy outcomes. The authors carefully selected the literature, meticulously reviewed the papers, and accurately reported the synthetic results.

Answer: We thank the reviewer for taking the time to review our work and recognize its merit!

Reviewer #3: This manuscript, "The impact of antenatal syphilis point of care testing on pregnancy outcomes: a systematic review" is a very thoughtful and comprehensive analysis of the impact of point of care syphilis tests on adverse pregnancy outcomes such as infant prematurity, stillbirth, and neonatal death. There is not a great deal of literature in this space, but the authors did an excellent job of summarizing what IS available. Their methodology is strong, and the write-up is clear.

Answer: We thank the reviewer for taking the time to review our work and recognize its merit!

My overall recommendations are really two-fold: consider shortening the Introduction section a bit (to get to the heart of your paper faster) and consider editing the Discussion section slightly to help the reader understand your main message more quickly (the main message I am seeing is: "each of these studies is flawed in its own way.")
Answer: We thank the reviewer for this suggestion. Introduction and discussion have been shortened as advised, we hope this is enough.

Below are my requests for minor revisions:
1) I found the number of acronyms in the manuscript to be overwhelming and to impede general comprehension. Some acronyms (e.g., POCT) are quite natural given their common use in this literature, but I found other acronyms (e.g., PM for prematurity, FL for fetal loss, NTT for non-treponemal test, ICS for ?) to be neither space-saving nor comprehension-improving. There is no need to abbreviate a one-word term like prematurity. There's also no advantage to introducing separate acronyms for STD and STI when one would likely suffice throughout. Please review all acronyms and reduce the total number to something more reasonable. An acronym is only advantageous when it saves a significant number of words AND when it doesn't require the reader to thumb through the manuscript looking for its original meaning.
Answer: We agree this is a needed improvement and following the reviewer’s advice, we reduced the number of acronyms in the text to improve readability. We hope the text reads better now.

2) It would be helpful if the authors introduced the dual POCT test as a 'screen and confirm' or 'non-treponemal and treponemal' dual test (at first use). The phrase 'dual test' is often used to reference HIV/syphilis combination tests, so the clarification might be helpful for a broader audience.

Answer: As suggested by the reviewer this has been revised in line 257.

3) The Introduction section feels quite long. I'm already a bit tired by the time I get to the most interesting parts of the manuscript--Methods & Results. I would recommend making some cuts in the Introduction to improve its punchiness and get to the 'so what?' of your work a little faster. For consideration: Cut the sentence beginning with "This initiative was followed by..." on Line 60. We don't need as much history about WHO's strategies to understand your work. Cut the sentences that give examples of traditional lab tests (i.e., TPPA, TPHA). Knowing the names of specific treponemal (bench) tests does not help me understand your point-of-care testing work any better. Cut the entire paragraph about the future of PCR testing. This paragraph is not needed to set up the importance of your POCT work. The sentence beginning on Line 107--which lists WHO's POCT requirements--feel very redundant to everything you already laid out in lines 103-106. Find a way to reduce this redundancy. Consider finding a way to introduce a paragraph break somewhere in lines 60-93 as well (suggested in Line 78) to help with the visual flow of that very long paragraph.
Answer: We thank the reviewer for having provided practical suggestions on how to improve the introduction. Those suggestions have been followed.

4) Comments about prevalence and case-count in the first paragraph of the Introduction need a qualifier (i.e., 'globally' or 'in the world') so the reader understands what geography you're referencing (Line 42 and 45).
Answer: This has been revised as suggested in lines 47 and 51.

5) Line 51: Early syphilis also includes early latent syphilis (not just primary and secondary). Please add to your parenthetical.

Answer: This has been revised in line 56.

6) Add a citation to Lines 52-53 (vertical transmission comment).

Answer: This has been revised in line 58.

7) Line 64: "fewer," not "less." (There are several instances of this throughout the manuscript that should be checked).
Answer: This has been revised throughout the text

8) Line 119 would read more clearly as: "Yet challenges remain in the implementation of POCT, particularly in resource-limited settings, such as acceptance by local healthcare workers..."

Answer: This has been revised in lines 253-256

9) Lines 130-134: This paragraph feels like it could be in the Discussion section. This is a strong recommendation (but not a requirement) that you consider relocating this paragraph. It seems to make more sense to tout the contributions (potential or real) of your work AFTER you've shared the results, not before.

Answer: We thank the reviewer for this suggestion, this has been followed.

10) Line 140: I had never heard/used the term 'meta-synthesis' before. It struck me as a little odd. If this term is preferred by PLOSOne, then I defer. Otherwise, consider using the phrase 'systematic literature review,' which is what this really is and will leave your readers with less head-scratching. If you'd like to keep the phrase 'meta-synthesis' then it might be helpful to provide some basic features of a meta-synthesis that make it different from a systematic literature review.
Answer: We thank the reviewer for this suggestion, this term has been removed.

11) Consider including treatment coverage in your list of 'predictors' in Lines 148-152.
Answer: This was indeed mistakenly left out, it has now been revised in line 333.

12) Line 159: "...low birth weight, prematurity, miscarriage, and stillbirth..."
Answer: This has been revised in line 342

13) Instead of saying 'up until June 8th,' it might be more appropriate to say that the search was conducted in 3 databases and included all literature published as of June 8th.
Answer: This has been revised in line 354

14) Line 179: "were screened" not "where screened."
Answer: This has been revised in line 370

15) Line 188: "...maternal and gestational age were extracted..."
Answer: This has been revised in line 385

16) Methods: Add a comment to your methods section (likely to last paragraph of Methods) that addresses whether the bias scores will be used for inclusion/exclusion purposes. It doesn't look like any studies were dropped because of moderate bias scores, but it might be helpful to have you comment as to why that is (or what's customary when using these bias scores).
Answer: Indeed the risk of bias was not used to exclude studies from the review. This is normal custom in systematic review, we added this in lines 400-401.

17) Line 246: Would it be appropriate to replace the phrase 'sampling' with 'first ANC visit?' This would read more intuitively if it's accurate. It reminds the reader that these are real tests whose use is intended to be 'screening at first ANC visit' in most places. If this is not an accurate description of how women were sampled, then you can ignore this recommendation.
Answer: this has been revised in line 451.

18) Lines 246-247: "et al" not "at al"
Answer: This has been revised in line 452.

19) Line 255: "four-fold higher than maternal titer"
Answer: This has been revised in line 467.

20) Line 282: Clarify what sensitivity and specificity you're referencing here. Is it sensitivity and specificity of only the POCT in question? Or were sensitivities and specificities of all comparators taken into consideration, too?
Answer: This has been clarified in lines 517-518.

21) Line 315-317: Did Bristow really posit an increase in adverse outcomes? Or it it more accurate to say that Bristow posited no difference in adverse outcomes? Your next paragraph makes it sound like the Bristow reference (#39) really showed no difference. If that's the case, I would tone down the language you use in lines 315-317.
Answer: This has been revised in line 576.

22) Lines 318: Please try to use a consistent number of decimal places as you're reporting findings in this paragraph. It makes sense that for cases you would have 0 decimal places, but for rates per 1,000 pregnancies, try to be consistent.
Answer: This has been revised throughout the section. When decimals are reported, we consistently used 2 after the comma, with the exception of the last , which needs three. We hope this contributes to better consistency.

23) Lines 381-402: The framing of this Discussion section is a little hard to follow. While reading the Results section, I clearly understood that the Bristow paper produced different results than all other modeling studies. I am not certain why the authors are leading with a discussion of the Kruznik papers before discussing Bristow, which seems like the real outlier? It might make the MOST sense to structure your Discussion by walking through model assumptions/inputs (one paragraph on sensitivity/specificity, one paragraph on treatment coverage, one paragraph on syphilis prevalence) and discussing how each paper made different choices leading to different outcomes.
Answer: We thank the reviewer for having provided practical suggestions on how to improve the discussion. As suggested, we are now leading with a discussion of Bristow’s study like suggested (from line 688). However, after careful consideration we feel that restructuring the discussion by input parameters used (one paragraph on sensitivity/specificity, one paragraph on treatment coverage, one paragraph on syphilis prevalence) would make it more difficult to follow for two reasons:

1. The differences in pregnancy outcomes are a result of the interplay of different model assumptions rather than of single input parameters (E.g Kuznik et al. assumed relatively low syphilis prevalence rates AND treatment rates were not given, which resulted in fewer averted adverse pregnancy outcomes).

2. Papers like the ones by Schackman, Blandford, Rydzak or Owusu-Edusei did model and compare the effects of several different types of POCTs in one study (treponemal, non-treponemal and dual POCTs). If the structure would be changed like suggested and each paper would be discussed by input parameters used, readers might lose the overview of the effects of individual tests, which in the end is the aim of this review.

Therefore, to highlight the differences of POCT types we structured the discussion according to test types rather than authors or input parameters. We included subheadings in the discussion in the hope that the discussion is now easier to follow.

24) Line 464: "...authors' prediction model..." It would also be helpful to refer to this as a treponemal-only POCT vs. a dual POCT in your comparison. ICS feels like an inappropriate term to use, because most dual POCTs are also ICS, right?
Answer: this has been revised in line 821.

25) Line 486: "...poor improvements in testing..." might be better stated as "...modest improvements in testing..." to improve understanding. A poor improvement is a tough oxymoron to interpret.
Answer: This has been revised in line 851.

26) Line 518: Is HIV testing coverage 40-50% higher or 40-50 percentage points higher? Based on my prior work, I suspect it's the latter, so check your word choice here to make sure you're saying what you mean.
Answer: This has been revised in line 886.

27) Line 535: "clinical studies"
Answer: This has been revised in line 908.

28) Line 536: Instead of "implemented in the model" consider: "...used to construct the model."
Answer: This has been revised in line 909.

29) Line 540: Clarify the distinction that you're trying to draw (i.e., some country- and region-level prevalence estimates were generated using treponemal-only methods, which would not indicate a measure of active syphilis infection, but rather a measure of past or current infection.)
Answer: This has been clarified in lines 915-916.

30) Line 547-548: I like the fact that you specify reasons why a 100% treatment rate is not a reasonable assumption; I would also add stockouts to the list of reasons. The supply chain for syphilis tests and treatments is not nearly as strong as the supply chain for HIV tests and treatments and stockouts are common, particularly due to lapses in funding for syphilis products. (HIV products rarely experience these lapses in funding).

Answer: This has been revised in line 941.

31) In your limitations section, I would like to see you add/explore the idea that sensitivity and specificity inputs for these modeling studies often use manufacturer-provided sensitivities/specificities, which are indicative of a lab atmosphere in the Western Hemisphere. Often times, 'real world' studies measuring sensitivity and specificity of rapid diagnostic tests used in the field (i.e., Bioline) show significantly lower test characteristics when used by REAL end-users than they do in a lab in Europe (i.e., the manufacturer's insert.) It does make me wonder what TRUE sensitivity/specificity of many of these POCTs would be in the field and how this may impact pregnancy outcome calculations.
Answer: This has been revised and a new section added to the limitations.

32) Line 572: "show" not "shown"

Answer: This has been revised in line 967.
